# Task-Irrelevant Novel Sounds Improve Attentional Performance in Children With and Without ADHD

**DOI:** 10.3389/fpsyg.2015.01970

**Published:** 2016-01-05

**Authors:** Jana Tegelbeckers, Laura Schares, Annette Lederer, Bjoern Bonath, Hans-Henning Flechtner, Kerstin Krauel

**Affiliations:** ^1^Department of Child and Adolescent Psychiatry and Psychotherapy, Otto von Guericke University MagdeburgMagdeburg, Germany; ^2^Center for Behavioral Brain SciencesMagdeburg, Germany

**Keywords:** ADHD, attention, novelty, distraction, flanker task

## Abstract

Task-irrelevant salient stimuli involuntarily capture attention and can lead to distraction from an ongoing task, especially in children with ADHD. However, there has been tentative evidence that the presentation of novel sounds can have beneficial effects on cognitive performance. In the present study, we aimed to investigate the influence of novel sounds compared to no sound and a repeatedly presented standard sound on attentional performance in children and adolescents with and without ADHD. We therefore had 32 patients with ADHD and 32 typically developing children and adolescents (8 to 13 years) execute a flanker task in which each trial was preceded either by a repeatedly presented standard sound (33%), an unrepeated novel sound (33%) or no auditory stimulation (33%). Task-irrelevant novel sounds facilitated attentional performance similarly in children with and without ADHD, as indicated by reduced omission error rates, reaction times, and reaction time variability without compromising performance accuracy. By contrast, standard sounds, while also reducing omission error rates and reaction times, led to increased commission error rates. Therefore, the beneficial effect of novel sounds exceeds cueing of the target display by potentially increased alerting and/or enhanced behavioral control.

## Introduction

Characterized by persisting levels of inattention, hyperactivity, and impulsivity, attention-deficit/hyperactivity disorder (ADHD) is one of the most common developmental disorders worldwide (Polanczyk et al., 2007). According to the diagnostic criteria of the DSM IV (American Psychiatric Association [APA], 1994), attentional impairments can be evident in lapses of attention, carelessness in cognitive tasks, forgetfulness, or increased distractibility. Posner and Petersen (1990) proposed an influential model that separates the complex construct of attention into three independent components for alerting, orienting/reorienting and executive control, all of which rely on distributed neural networks (Fan et al., 2005). Alerting refers to obtaining an alert state during a task that is necessary for sustaining attention and preventing recurrent lapses of attention. Orienting/Reorienting toward a task is required when sensory stimulation outside the current attentional focus involuntarily attracts attention. Finally, executive control is crucial to resolve response conflicts. In imaging studies, children with ADHD show alterations in all of these three attentional networks (Konrad et al., 2006). Additionally, impairments in behavioral measures that rely on these networks have been shown in ADHD in numerous tasks such as continuous performance tasks examining sustained attention (Huang-Pollock et al., 2012), stop and go-nogo tasks that investigate response inhibition (Lijffijt et al., 2005), or Flanker and Simon tasks assessing interference control (Mullane et al., 2009).

However, ADHD patients do not consistently show impairments in attentional tasks (Huang-Pollock and Nigg, 2003) and various studies have identified specific task conditions or stimulus features that can normalize attentional functioning in ADHD. For instance, reinforcement could improve response inhibition (Konrad et al., 2000; Slusarek et al., 2001) and higher task difficulty, requiring increased alertness, normalized the ability to filter distractors in a visual paradigm in children with ADHD (Friedman-Hill et al., 2010). Furthermore, ongoing background stimulation can have a beneficial influence on ADHD patients: arithmetic task performance benefitted from background-music (Abikoff et al., 1996), white noise has been shown to improve free recall performance (Söderlund et al., 2007), and hyperactive symptoms decreased in a waiting situation when visual stimulation was provided (Antrop et al., 2000). In addition to such concurrent stimulation, the influence of interspersed and particularly of novel auditory stimuli has received interest. Novelty is a potent feature that elicits an automatic orienting response (Sokolov, 1963) and can positively influence executive control processes by activating the orienting network (Fan et al., 2005). This mechanism is important in case of unexpected events (e.g., an alarm or flashing light) that might require behavioral adaptation. However, attentional orienting toward sounds can also have a detrimental effect on performance, typically by delaying subsequent responses (for a review see Parmentier, 2014), especially in children (Wetzel and Schröger, 2007). In earlier studies, it was therefore expected that ADHD patients would particularly suffer from the presentation of irrelevant novel sounds during cognitive tasks due to their distractibility and poor cognitive control. Indeed, Gumenyuk et al. (2005) showed that in ADHD patients auditory novel stimuli increased the number of omission errors in a simple visual decision task. This was accompanied by alterations in the electro-physiological components associated with novelty processing (early and late P3a).

Although the electrophysiological evidence for an increased attention switch to novel stimuli in ADHD is inconsistent (for a review see Barry et al., 2003), we found in a recent fMRI study, focusing on the neural representation of novelty in ADHD, that task-irrelevant novel pictures increased activity in areas related to attention orienting and semantic analysis in ADHD patients compared to typically developing children and adolescents (Tegelbeckers et al., 2015). These findings suggest that behaviorally irrelevant novel stimuli are more likely to distract patients with ADHD.

Interestingly, van Mourik et al. (2007) found first evidence that task-unrelated environmental novel sounds might improve accuracy in a visual two-choice reaction time task in comparison to a standard tone (600 Hz), particularly in children with ADHD. Their results indicated a facilitating effect of novelty on task performance, which could be brought upon by activating the attentional orienting network. However, their design did not allow one to address whether task-irrelevant novel sounds do indeed have a facilitating effect, because task performance without preceding auditory stimulation was not assessed. Moreover, novel sounds were at the same time more meaningful (environmental vs. artificial sound) and less frequent than the standard tones questioning whether novelty was the crucial beneficial feature of task-irrelevant stimulation. Finally, typically developing children performed very close to ceiling, thus it is yet unclear whether ADHD patients are particularly responsive to novelty.

Because the identification of advantageous task-stimulus constellations is of utter importance and high clinical relevance for children and adolescents with ADHD, we aimed to investigate more thoroughly the effect of task-irrelevant novel sounds on attentional performance in ADHD. We decided to use a flanker paradigm that has been reported to lead to sufficiently high error rates in children with and without ADHD (Mullane et al., 2009). Thereby, we hoped to overcome potential ceiling effects, particularly in the comparison group that previously might have prevented modulations of novel sounds to appear (Gumenyuk et al., 2005; van Mourik et al., 2007). Moreover, the flanker task allows one to investigate the influence of sounds not only on sustained attention, as in simple visual decision tasks, but also on interference control evident in the degree of performance deterioration due to the incongruent flanking stimuli. We also introduced a no-sound condition to assess facilitating and distracting effects of both sounds. To furthermore separate novelty from rarity, we presented novel sounds with the same probability as the standard sound and the no-sound condition. Finally, we wanted to overcome differences in stimulus salience between novel and standard tones. Therefore, both categories were chosen from a pool of meaningful environmental sounds.

Based on van Mourik et al.’s (2007) previous results, we expected that children in both groups would show lower error rates in the sound conditions compared to the no-sound baseline. This improvement was also expected to be greater for novel than standard sounds and more pronounced in children with ADHD than in the healthy comparison group.

## Materials and Methods

### Participants

Overall, 72 children aged between 8 and 13 years participated in this study. They were mainly recruited through advertisements in the local newspaper or referred to us by the Department of Child and Adolescent Psychiatry and Psychotherapy. During the diagnostic procedure, trained interviewers supervised by experienced child and adolescent psychotherapists carried out in-person interviews separately with all parents and all participants above the age of ten. The German adaptation (Delmo et al., 2000) of the Revised Schedule for Affective Disorders and Schizophrenia for School-Age Children: Present and Lifetime Version (K-SADS-PL; Kaufman et al., 1997) was used to assess clinical symptoms according to DSM IV. Moreover, the Child Behavior Checklist (CBCL, Achenbach, 1991a) and the Youth Self Report (YSR, Achenbach, 1991b) were employed for additional clinical evaluation. These questionnaires assess internalizing and externalizing critical behavior and provide normed scores for clinically relevant syndromes (*T* > 65).

The diagnostic criteria for ADHD according to DSM IV as assessed by the K-SADS-PL were met by 31 boys and five girls (mean age: 10.61 ± 1.61). Among them, 29 participants were diagnosed with the combined subtype of ADHD, six with the primarily inattentive and one with the hyperactive/impulsive subtype. Participants with stimulant medication (*N* = 18) discontinued the intake at least 24 h before and on the day of the experiment. The comparison group consisted of 31 boys and five girls (mean age: 10.58 ± 1.71) considered typically developing based on the diagnostic interview, questionnaire results, and test performance. ADHD patients who met present or lifetime criteria for any psychiatric disorder other than oppositional defiant disorder (ODD, *n* = 12) or conduct disorder (CD, *n* = 1) were excluded from the sample. Control participants were excluded if there was evidence of any previous or current psychiatric disorder. Furthermore, exclusion criteria for all subjects included the existence of hearing impairments, an IQ below 80, or evidence for substance abuse. All participants had normal or corrected-to-normal vision.

Intelligence was assessed with the German adaptation of the Culture Fair Intelligence Test Scale 20 (CFT-20-R; Weiss, 1997) and attentional performance was evaluated by the d2 – Attention Endurance Test (d2; Brickenkamp, 2002). For children below the age of nine (*N* = 10) the age-adjusted versions of both tests were used (CFT-1, Cattell et al., 1995; bp-test, Esser et al., 2008). Moreover, the Verbal Learning and Memory Test (VLMT, Helmstädter et al., 2001) was administered to account for verbal encoding and immediate recall, delayed recall, and recognition deficits.

As shown in **Table 1**, patients and typically developing participants showed no significant differences in age, but differed significantly when tested for their IQ. However, as groups were not randomly assigned, such differences were to be expected as ADHD is often associated with lower IQ values (Kuntsi et al., 2004). Furthermore, groups differed significantly in their attentional performance (d2) and self-and-proxy assessment of attention related problems (YSR, CBCL). Memory measures of the VLMT showed a significant difference between groups with regards to learning, delayed recall, and recognition.

**Table 1:** Characteristics of the ADHD sample and the comparison group of typically developing children (TD).

	ADHD	TD	*t*
Measure	Mean (±*SD*)	Mean (±*SD*)	(paired)
Gender	31 male, 5 female	31 male, 5 female	
Age	10.61 (±1.61)	10.58 (±1.71)	0.71
IQ (CFT-20-R/CFT-1)	101.92 (±13.76)	110.08 (±11.97)	2.69∗∗
Attentional Performance (d2/bp-test)	54.36 (±30.46)	74.56 (±24.42)	3.10∗∗
Verbal Learning and Memory Test			
- Learning	48.89 (±10.44)	54.2 (±8.93)	2.3∗
- Delayed recall	50.38 (±8.87)	55.13 (±8.32)	2.33∗
- Recognition	48.74 (±10.2)	55.71 (±10.82)	2.77∗∗
Attentional Problems (self-rating, YSR)	60.42 (±8.44)	53.00 (±5.11)	7.62∗∗∗
Attentional Problems (parental rating, CBCL)	67.71 (±7.06)	55.10 (±5.34)	3.23∗∗

*∗*p* < 0.05, ∗∗*p* < 0.01, ∗∗∗*p* < 0.001*.

All participants and their parents received detailed information about the study and gave written assent/consent. Children and adolescents received 5€, per hour in the form of gift vouchers. The study was approved by the local ethics committee of the University of Magdeburg, Faculty of Medicine, and followed the ethical standards of the Declaration of Helsinki.

### Task and Procedure

**FIGURE 1:**
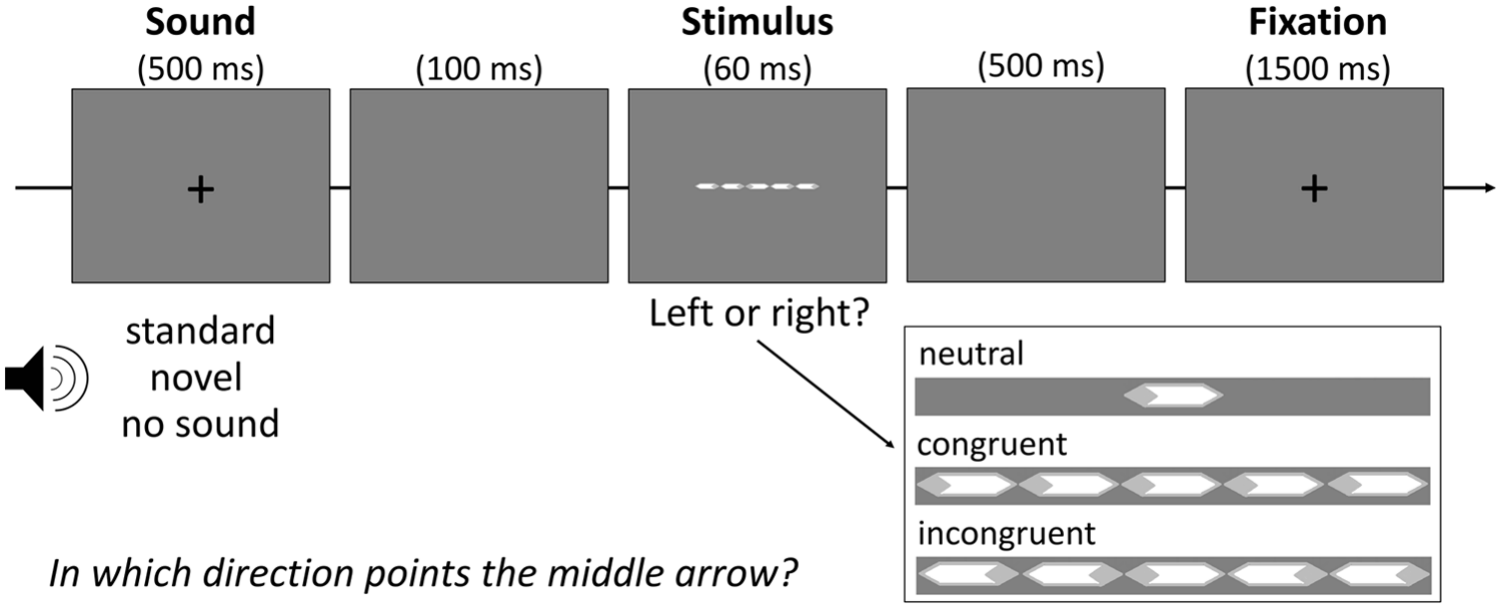
**Schematic illustration of the modified flanker task**.

To assess attentional performance in this study, we chose an adaptation of the Eriksen flanker task (Eriksen and Eriksen, 1974) that consisted of white arrows masked by a light-gray rhombus on gray background (see **Figure 1**). Participants had to indicate the direction of the target arrow in the center of the screen via button press. In line with previous studies, this target arrow appeared either alone (neutral flanker condition) or flanked by two simultaneously presented arrows on each side. These flanking arrows pointed into either the same direction as the target arrow (congruent flanker condition) or the opposite direction (incongruent flanker condition). Each condition (neutral, congruent, incongruent) was presented with equal frequency of 48 times.

Randomly intermixed with a baseline without sound stimulation, two-thirds of all trials per condition were preceded by a sound. In half of these trials (*N* = 48) the sound was a repeatedly presented sound serving as standard. In the other half, novel nonrecurring sounds were presented. A pool of 140 auditory stimuli of environmental content (e.g., dog bark, doorbell) was selected for this study from a German commercial CD (“1.111 Geräusche”, Döbeler Cooperations, Hamburg, Germany). All sounds were edited with the software audacity (www.audacity.sourceforge.net) to be of equal volume (60 dB) and duration (500 ms). Then, 49 sounds were randomly assigned to every participant: one to serve as the standard and 48 novels. All sounds were presented over headphones.

During task instruction, participants were informed that sounds would be presented throughout the experiment, but that they were unrelated to the task and could be ignored. The experiment started with a training run of 12 trials in order to familiarize the participants with the task and the standard sound. Subsequently, two experimental runs of the flanker task were carried out with a short break halfway through. Overall, the experiment lasted approximately 15 min and was carried out on a laptop with a 17.3 inch screen and Presentation software (Version 16.0, www.neurobs.com).

As **Figure 1** shows, every trial started with the simultaneous presentation of a black fixation cross and the auditory stimulus for 500 ms, followed by a blank screen for 100 ms. Right after the display of the arrows (60 ms), a blank screen was shown again for 500 ms followed by the presentation of another fixation cross for 1500 ms. Participants were instructed to be as fast and accurate as possible.

### Data Analysis

Attentional performance was assessed via percentage of errors (commission and omission) as well as mean reaction time (mRT) and reaction time variability (RTV) for correct trials. The latter was computed by individually normalizing the standard deviation with the mean reaction time (SD/mRT). Furthermore, the flanker effect as a measure of interference control was computed by subtracting performance in congruent trials from incongruent trials for mean reaction times, RTV, and error rates, respectively. Participants whose performance in overall error rate or mean RT differed for more than two standard deviations from their respective group mean were excluded from further analysis. This led to a final sample size of *N* = 64 (five girls per group). However, sample characteristics did not differ from the ones previously reported.

In the statistical analyses, we carried out 2x3 repeated-measures analyses of variance (ANOVA) on error rates, mean reaction times, and reaction time variability, including the factors *group* (ADHD vs. TD) and *sound* (standard vs. novel vs. no sound). All results are summarized in **Table 2**. To account for violations of sphericity, the Greenhouse-Geisser correction was applied if necessary. The results from the ANOVAs were further investigated by *post hoc t*-tests, if applicable. Finally, we correlated all performance measures with IQ separately for each group.

**Table 2:** Overview over the 2 × 3 ANOVAs including *group* (ADHD vs. TD) and *sound* (standard vs. novel vs. no sound).

	Main Effect *Group*		Main Effect *Sound*		Interaction Effect	
	*F* (p)	η_*p*_^2^	*F* (p)	η_*p*_^2^	*F* (p)	η_*p*_^2^
Commission error rate	12.83∗∗	0.172	8.45∗∗	0.12	0.17 (0.819)	0.003
Omission error rate	14.47∗∗∗	0.189	11.99∗∗∗	0.162	1.21 (0.29)	0.019
Mean reaction time	3.79 (0.056)	0.058	51.65 ∗∗∗	0.454	2.28 (0.115)	0.035
Reaction time variability	13.21∗∗	0.176	3.53∗	0.054	0.69 (0.502)	0.011

*Significant effects are flanked by ∗*p* < 0.05, ∗∗*p* < 0.01, ∗∗∗*p* < 0.001*.

*Effect sizes are provided in form of η_*p*_*^2^.

## Results

**FIGURE 2:**
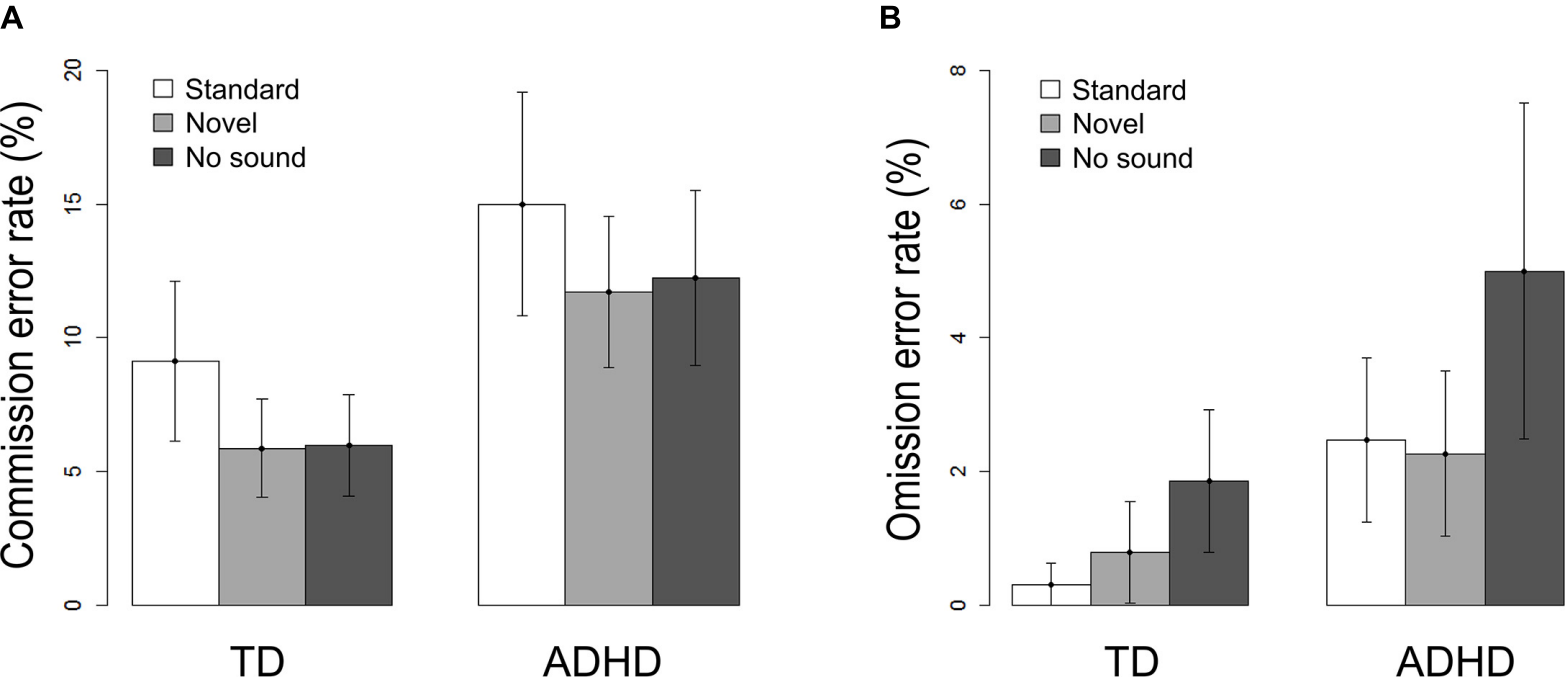
**Task performances per sound condition and per group separated for **(A)** Commission errors and **(B)** Omission errors**. Error bars denote confidence intervals of 95%.

Accuracy in the flanker task is described by the percentage of commission errors (false responses) and omission errors (misses). **Figure 2** shows the influence of the sound conditions on both error rates per group. The 2×3 ANOVAs revealed main effects of *group* and *sound*: Children with ADHD performed less accurately than the comparison group regarding the commission error rate (*Fgroup*_(1,62)_ = 12.83, *p* = 0.001) as well as omission error rate (*Fgroup*_(1,62)_ = 14.47, *p* < 0.001). However, the modulatory influence of sound differed between the types of errors. Concerning commission errors, the main effect of sound [*F*_(1.8,124)_ = 8.45, *p* = 0.001] resulted from a significant decrease in performance following the standard sound compared to novel sounds [*t*_(63)_ = 3.25, *p* = 0.002] or trials with no sound stimulation [*t*_(63)_ = −2.63, *p* = 0.011] in both groups. For omissions, both sound conditions led to a significant improvement compared to trials without a sound [*Fsound*_(1.36,124)_ = 11.99, *p* < 0.001; standard: *t*_(63)_ = −3.29, *p* = 0.002; novel: *t*_(63)_ = −3.16, *p* = 0.002].

**FIGURE 3:**
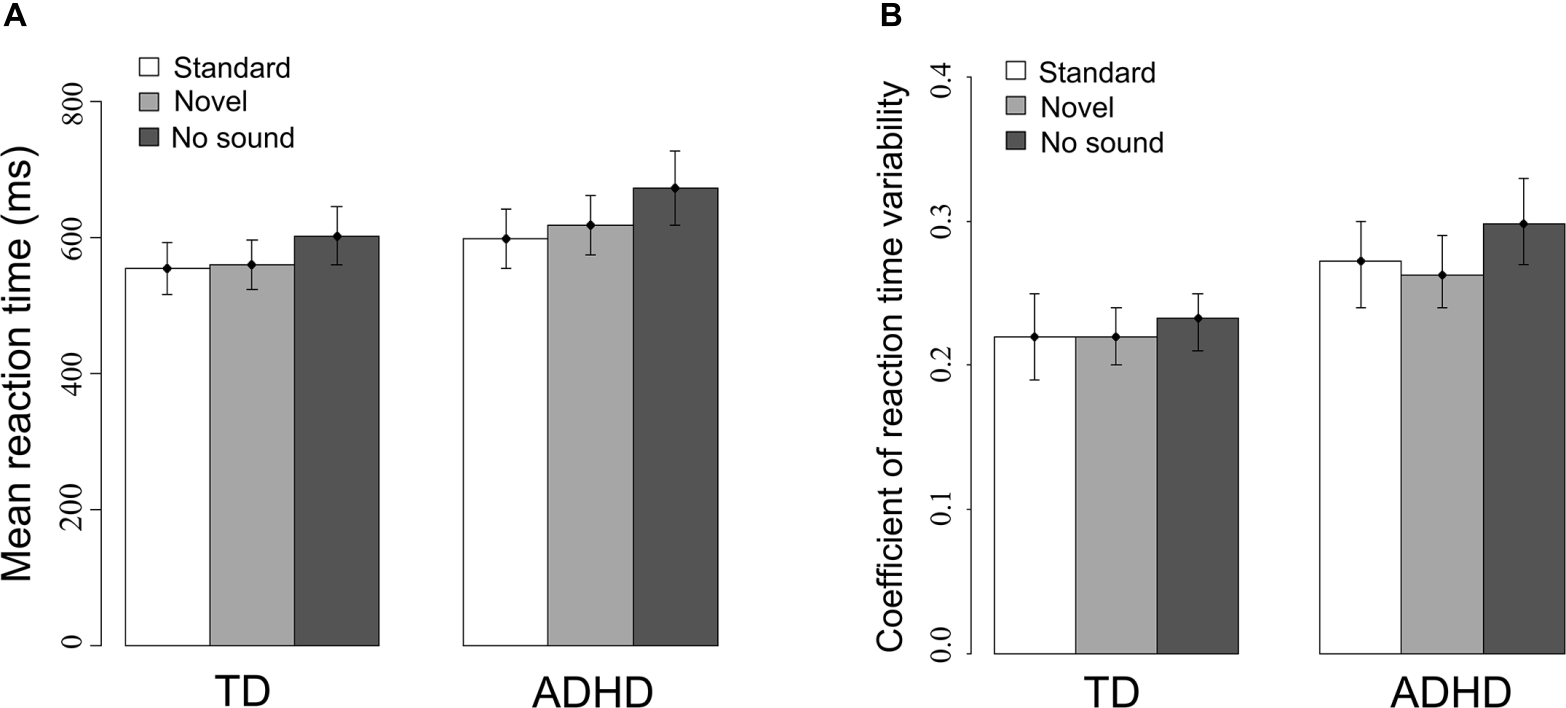
**(A)** Mean reaction times and **(B)** reaction time variability (SD/mean) with 95% confidence intervals for both experimental groups for all three sound conditions.

Similarly, the means and variabilities of reaction time were modulated by *sound* [mRT: *F*_(1.71,124)_ = 51.65, *p* < 0.001; RTV: *F*_(2,124)_ = 3.53, *p* < 0.05] as depicted in **Figure 3**. Mean RT decreased in trials with a sound compared to the no-sound baseline [standard: *t*_(63)_ = −6.9, *p* < 0.001; novel: *t*_(63)_ = −4.92, *p* < 0.001] and was shorter when the standard sound was presented compared to novel sounds [*t*_(63)_ = −2.38, *p* = 0.02]. Furthermore, novel sounds [*t*_(63)_ = −2.85, *p* < 0.01] but not standard sounds [*t*_(63)_ = −1.81, *p* = 0.08] reduced RTV compared to trials without preceding sound. Group differences were only observed for RTV indicating that ADHD patients were significantly more variable in their reaction times than typically developing children [*Fgroup*_(1,62)_ = 13.21, *p* < 0.01].

None of the presented results changed when children with inattentive subtype or comorbid dissocial disorders were excluded from the analysis.

The analyses of the flanker effect (difference value: incongruent-congruent) for mean RT, RTV, and error rates revealed no influence of sound nor group on interference control. Finally, none of the performance measures correlated significantly with IQ (all *r* < 0.4).

## Discussion

The current study aimed to investigate the influence of task-irrelevant sounds on attentional performance of children and adolescents with and without ADHD during a flanker task. In particular, we were interested in the performance modulation by unique novel sounds compared to a repeatedly presented standard sound and a baseline without sound presentation. We found that both sounds improved reaction times and omission error rate compared to no sound but only novels also reduced reaction time variability. Moreover, standard but not novel sounds increased the commission error rate.

As expected from previous research on the flanker task (Mullane et al., 2009), children with ADHD performed worse than the comparison group regarding accuracy measures and reaction times. This could be associated with difficulties in interference control (Mullane et al., 2009), sustained attention (Huang-Pollock et al., 2012), or behavioral control (Lijffijt et al., 2005). However, although they performed better than the ADHD patients in this study, the group of typically developing children produced error rates between 6 and 10%, indicating that the task was difficult enough to reveal sound modulations also in the comparison group. This resolved a potential limitation of previous studies, because beneficial effects of sounds on task performance can only unfold when performance is not at ceiling (van Mourik et al., 2007; Alderson et al., 2008). Indeed, we found performance modulations by sounds not only on reaction times and reaction time variability but also on error rates in healthy children as well as in children with ADHD. No interaction effects could be detected, indicating more similar processing in both groups than expected before (Gumenyuk et al., 2005; van Mourik et al., 2007). The sounds influenced both groups with comparable magnitude and in the same direction, which is in line with the recent finding of similar neural activations during the processing of novel stimuli in children with and without ADHD (Tegelbeckers et al., 2015).

Insufficient task performance in the flanker task is composed of two different types of errors that are based on different processes: missed responses follow lapses of attention and false reactions occur due to failure in behavioral control: namely, in interference control, the suppression of task-irrelevant competing stimuli, and in response inhibition, the suppression of a pre-potent response. Considering omission errors, we observed a beneficial effect of both sound conditions compared to the no-sound condition, indicating that they both served as alerting signals. The sounds announced the task display, functioning as cues, and enabled response preparation likewise in children with and without ADHD. Previous research has already shown that the impact of a task irrelevant sound depends more largely on the informational value it transmits than on the content (Parmentier et al., 2010) and that children with ADHD can benefit from meaningful sounds in the same way as typically developing children (Alderson et al., 2008; Mullane et al., 2011).

However, although both sound conditions in our experiment were of identical informational value regarding the onset of the target display, we observed differential effects on commission errors, reaction times, and reaction time variability. Standard compared to novel sounds increased the commission error rate in both groups and decreased reaction times. These results suggest a speed accuracy trade-off similar to the findings by van Mourik et al. (2007): the repeated presentation of the standard sound might have favored the initiation of pre-potent responses thereby increasing the probability of mistakes. Novel sounds, on the other hand, also accelerated responses compared to the no-sound condition but apparently led to more thorough processing of task relevant stimuli than the standard sound. Similar effects of prolonged reaction times by novel sounds have been shown before (Wetzel et al., 2012) and can be attributed to attentional orienting toward incoming unexpected information. The necessity to reorient attention could lead to slower processing of the task display *(orienting costs)* but also cause an increased alertness (*alerting benefits*; San Miguel et al., 2010). This is supported by electrophysiological findings from comparable paradigms where children and adults showed larger P3a amplitudes towards novel task-irrelevant stimuli without behavioral costs (van Mourik et al., 2007; Ruhnau et al., 2010; Wetzel et al., 2013).

Moreover, intra-individual variability of reaction times to the task relevant stimulus was only successfully reduced by preceding novel sounds. This is particularly relevant, because elevated RTV in cognitive tasks is one of the most stable characteristics in ADHD (Leth-Steensen et al., 2000; Vaurio et al., 2009; Tamm et al., 2012). In previous studies, beneficial effects on RTV in ADHD have been reported for reward, increased event rate, and stimulant medication (for review see Tamm et al., 2012). In our setup, novel sounds seemed to improve behavioral monitoring compared to the no-sound condition, actually suggesting a potential facilitating role of novel sounds in cognitive tasks. However, in contrast to studies employing reward, the novel sounds did not normalize RTV in the patient group to the level of typically developing children.

Furthermore, it is worth noting that novel sounds did not decrease the commission error rate compared to the no-sound trials, indicating that when trials were successfully attended, performance of the task was equally accurate in trials with no and novel sounds. However, the latter accelerated the response compared to the no-sound baseline suggesting more efficient processing of the target stimulus and/or more efficient response selection.

The flanker effect, which can be seen in deterioration of accuracy and reaction times in the incongruent compared to congruent condition, was not modulated by the sounds. Thus, there was no specific beneficial effect on interference control which is in line with previous studies showing that the influence of novel sounds is independent of the task’s cognitive demands (Parmentier, 2014). We therefore think that the sounds did not influence cognitive execution of the task per se but rather improved the amount of attentional resources allocated to the task relevant stimulus.

Overall, task accuracy as a combination of sustained attention and correct execution benefitted from the presentation of novel compared to standard and no sounds in both groups. Novelty is a salient feature that induces unspecific activation and attracts attention (Näätänen, 1992) because of its potential environmental relevance. Past studies already showed that orienting reactions following novel stimuli in ADHD seem to be intact (Kemner et al., 1996; Jonkman et al., 2000; Mullane et al., 2011; Tegelbeckers et al., 2015). However, due to deficits in the alerting and executive attention networks (Konrad et al., 2006; Mullane et al., 2011) the influence of novel sounds might be particularly relevant for individuals with ADHD. According to Posner and Petersen’s (1990) model on executive functioning, novel stimuli can activate the alerting but also the orienting network (Fan et al., 2005). Therefore, different explanations for the impact of novel sounds are possible.

Firstly, various models of ADHD pathogenesis have been proposing that distractibility as well as hyperactive behavior could serve to compensate for cortical hypoarousal (e.g., optimal stimulation theory, Zentall and Zentall, 1983; Sergeant, 2005). The cognitive-energetic model (CEM) (Sergeant, 2005) sees dysregulation of effort (motivation), arousal, and activation as key factors in ADHD interacting with attentional and executive problems. For example, arousal is supposedly evident in phasic responses during stimulus processing and can be increased by signal intensity or novelty whereas activation is associated with tonic physiologic readiness signifying alertness. The CEM predicts that the level of arousal/activation that is needed to achieve optimal performance is higher in individuals with ADHD than in healthy controls. This fits our results in the way that novelty could have induced arousal and thereby provided patients with a temporarily optimized activation level at which better cognitive functioning, reflected in improved overall performance, was possible. However, in contrast to investigations using concurrent background stimulation during task execution (Abikoff et al., 1996; Söderlund et al., 2007), our setup might not increase activation permanently but specifically for trials in which novel sounds appeared. Furthermore, the beneficial effect of meaningless noise might rather be based on auditory masking and operate over screening out distracting influences.

On the other hand, task related beneficial effects of novelty may also stem from the disruption of the ongoing activity initiated by the orienting network that enhanced executive control (Fan et al., 2005) or by activation of the anterior cingular cortex and insula. The latter structures are not only involved in novelty processing but are also responsible for switching from default mode activation to task positive networks (Seeley et al., 2007; Sridharan et al., 2008; Sripada et al., 2014). As children with ADHD are expected to suffer from poor regulation of the default mode network (Sonuga-Barke and Castellanos, 2007; Fassbender et al., 2009), increased involvement of these structures might also explain the behavioral benefit, especially the decreased reaction time variability. Future electrophysiological and/or imaging studies will have to clarify whether beneficial effects of task-irrelevant novelty are based on increased unspecific alerting, activation of the orienting network, or default mode network suppression.

However, our results of improved performance by task preceding novelty in children with and without ADHD contradict previous studies. In ADHD, distraction has been shown by the inclusion of neutral sounds or visual stimuli (Gumenyuk et al., 2005; Berger and Cassuto, 2014). Also in attentive children, distraction in terms of reaction time prolongation by novel sounds could have been expected (Wetzel and Schröger, 2014). However, former studies differ from ours in some aspects. First, we did not use a simple decision task but received moderate error rates in both groups. This might be important because the optimal stimulation level for an ongoing task might follow an inverted U-shape. When typically developing children perform at their optimum, additional stimulation might have a detrimental effect on specific cognitive functions (Helps et al., 2014). Moreover, the effect of novel sounds might differ inter-individually from beneficial to distracting. Secondly, novel sounds in our experiment occurred with the same probability as the standard sound, making their appearance much more predictable than in the usual oddball scenario (80% standard, 20% novel). Wetzel and Schröger (2007) already showed that the distraction by deviant sounds is reduced with the predictability of their occurrence. Therefore, previous findings might be biased by interacting effects of novelty and rarity. Furthermore, deceleration following the novel sound compared to the standard sound is frequently understood as evidence of distraction. In children with ADHD, however, slowing down could actually improve adaptive action selection, because these patients usually suffer from impulsive, variable reactions. However, without thorough understanding of the underlying neural mechanisms of the effect of particularly novel sounds on task performance the inconsistent findings are hard to resolve. Further research using imaging techniques and including children with varying degrees of attentional abilities is needed to better understand the bidirectional role of novelty in attention control.

Summarizing, our study shows that task-irrelevant novel sounds can facilitate attentional performance in children with and without ADHD indicated by reduced omission error rates, reaction times, and reaction time variability without compromising performance accuracy. These findings encourage to exploring practical applications of task irrelevant novel stimulation in homework or classroom settings to improve attentional performance in ADHD.

## Conflict of Interest Statement

The authors declare that the research was conducted in the absence of any commercial or financial relationships that could be construed as a potential conflict of interest.
